# Supplementary material for: Modeling glioblastoma heterogeneity as a dynamic network of cell states
Source: Mol Syst Biol. 2021 Sep 16;17(9):e10105. doi: 10.15252/msb.202010105 (PMC8444284; doi:10.15252/msb.202010105)
Supplement: Supplementary file 6 — Source Data for Figure 5 [file MSB-17-e10105-s004.zip › Figure5A_sourcedata/GSEA_3017/nosigdb_stateF.GseaPreranked.1622795015644/DOROSZKO_U3013MG_CLUSTER_2.html]

Details for gene set DOROSZKO\_U3013MG\_CLUSTER\_2[GSEA]

|  || Dataset | state33017 |
| Phenotype | NoPhenotypeAvailable |
| Upregulated in class | na\_neg |
| GeneSet | DOROSZKO\_U3013MG\_CLUSTER\_2 |
| Enrichment Score (ES) | -0.8643917 |
| Normalized Enrichment Score (NES) | -6.164258 |
| Nominal p-value | 0.0 |
| FDR q-value | 0.0 |
| FWER p-Value | 0.0 |
Table: GSEA Results Summary

  

Fig 1: Enrichment plot: DOROSZKO\_U3013MG\_CLUSTER\_2      
 Profile of the Running ES Score & Positions of GeneSet Members on the Rank Ordered List

  

| PROBE | GENE SYMBOL | GENE\_TITLE | RANK IN GENE LIST | RANK METRIC SCORE | RUNNING ES | CORE ENRICHMENT || 1 | CAPS |  |  | 573 | 0.407 | -0.2732 | No |
| 2 | SYPL1 |  |  | 1137 | 0.287 | -0.5423 | No |
| 3 | KHDRBS1 |  |  | 1573 | -0.278 | -0.7499 | No |
| 4 | CDKN2C |  |  | 1593 | -0.285 | -0.7571 | No |
| 5 | HP1BP3 |  |  | 1618 | -0.290 | -0.7666 | No |
| 6 | FAM83D |  |  | 1624 | -0.292 | -0.7669 | No |
| 7 | APOLD1 |  |  | 1648 | -0.299 | -0.7759 | No |
| 8 | OIP5 |  |  | 1686 | -0.314 | -0.7915 | No |
| 9 | G2E3 |  |  | 1687 | -0.315 | -0.7893 | No |
| 10 | CENPQ |  |  | 1711 | -0.322 | -0.7981 | No |
| 11 | KIF20A |  |  | 1792 | -0.358 | -0.8342 | No |
| 12 | POC1A |  |  | 1794 | -0.360 | -0.8321 | No |
| 13 | ILF2 |  |  | 1795 | -0.360 | -0.8296 | No |
| 14 | NEK2 |  |  | 1815 | -0.371 | -0.8361 | No |
| 15 | SAP30 |  |  | 1826 | -0.378 | -0.8383 | No |
| 16 | MASTL |  |  | 1843 | -0.385 | -0.8433 | No |
| 17 | ACTL6A |  |  | 1859 | -0.393 | -0.8477 | No |
| 18 | EXOSC8 |  |  | 1865 | -0.397 | -0.8474 | No |
| 19 | TEDC1 |  |  | 1883 | -0.410 | -0.8527 | No |
| 20 | SHCBP1 |  |  | 1886 | -0.412 | -0.8507 | No |
| 21 | ASF1B |  |  | 1897 | -0.419 | -0.8526 | No |
| 22 | NSD2 |  |  | 1900 | -0.425 | -0.8506 | No |
| 23 | MXD3 |  |  | 1905 | -0.428 | -0.8495 | No |
| 24 | HNRNPH3 |  |  | 1911 | -0.429 | -0.8489 | No |
| 25 | MNS1 |  |  | 1913 | -0.430 | -0.8464 | No |
| 26 | DIAPH3 |  |  | 1924 | -0.439 | -0.8481 | No |
| 27 | DDX39A |  |  | 1957 | -0.458 | -0.8603 | No |
| 28 | ZWILCH |  |  | 1962 | -0.461 | -0.8590 | No |
| 29 | PTGES3 |  |  | 1970 | -0.467 | -0.8591 | No |
| 30 | POP7 |  |  | 1981 | -0.474 | -0.8605 | No |
| 31 | PGP |  |  | 1990 | -0.482 | -0.8610 | Yes |
| 32 | PHIP |  |  | 1992 | -0.483 | -0.8581 | Yes |
| 33 | SPAG5 |  |  | 1997 | -0.490 | -0.8566 | Yes |
| 34 | FAM111A |  |  | 2002 | -0.497 | -0.8550 | Yes |
| 35 | TK1 |  |  | 2003 | -0.497 | -0.8515 | Yes |
| 36 | XPO1 |  |  | 2007 | -0.500 | -0.8494 | Yes |
| 37 | CDK5RAP2 |  |  | 2012 | -0.503 | -0.8478 | Yes |
| 38 | HIRIP3 |  |  | 2017 | -0.508 | -0.8462 | Yes |
| 39 | FEN1 |  |  | 2018 | -0.511 | -0.8426 | Yes |
| 40 | RRM1 |  |  | 2023 | -0.516 | -0.8409 | Yes |
| 41 | CACYBP |  |  | 2028 | -0.518 | -0.8392 | Yes |
| 42 | PARPBP |  |  | 2030 | -0.520 | -0.8360 | Yes |
| 43 | JPT1 |  |  | 2036 | -0.525 | -0.8347 | Yes |
| 44 | CDCA3 |  |  | 2041 | -0.529 | -0.8329 | Yes |
| 45 | TRIP13 |  |  | 2044 | -0.530 | -0.8302 | Yes |
| 46 | TROAP |  |  | 2046 | -0.533 | -0.8269 | Yes |
| 47 | TTK |  |  | 2047 | -0.533 | -0.8232 | Yes |
| 48 | NCAPD3 |  |  | 2051 | -0.537 | -0.8208 | Yes |
| 49 | KIF22 |  |  | 2053 | -0.541 | -0.8175 | Yes |
| 50 | CKLF |  |  | 2055 | -0.544 | -0.8142 | Yes |
| 51 | SPDL1 |  |  | 2064 | -0.569 | -0.8140 | Yes |
| 52 | ZWINT |  |  | 2069 | -0.577 | -0.8119 | Yes |
| 53 | RTKN2 |  |  | 2071 | -0.579 | -0.8083 | Yes |
| 54 | RACGAP1 |  |  | 2072 | -0.579 | -0.8042 | Yes |
| 55 | CENPA |  |  | 2073 | -0.586 | -0.8001 | Yes |
| 56 | MND1 |  |  | 2074 | -0.586 | -0.7960 | Yes |
| 57 | SKA3 |  |  | 2077 | -0.590 | -0.7928 | Yes |
| 58 | BUB3 |  |  | 2078 | -0.596 | -0.7886 | Yes |
| 59 | AURKB |  |  | 2079 | -0.598 | -0.7844 | Yes |
| 60 | GGH |  |  | 2080 | -0.603 | -0.7802 | Yes |
| 61 | CENPK |  |  | 2082 | -0.607 | -0.7764 | Yes |
| 62 | USP1 |  |  | 2084 | -0.612 | -0.7726 | Yes |
| 63 | KNSTRN |  |  | 2085 | -0.615 | -0.7682 | Yes |
| 64 | CKAP5 |  |  | 2086 | -0.623 | -0.7639 | Yes |
| 65 | PLK1 |  |  | 2087 | -0.624 | -0.7595 | Yes |
| 66 | NCAPD2 |  |  | 2089 | -0.627 | -0.7556 | Yes |
| 67 | DNAJC9 |  |  | 2097 | -0.641 | -0.7544 | Yes |
| 68 | RFC3 |  |  | 2098 | -0.642 | -0.7499 | Yes |
| 69 | PHF19 |  |  | 2101 | -0.650 | -0.7463 | Yes |
| 70 | ITGB3BP |  |  | 2102 | -0.652 | -0.7417 | Yes |
| 71 | NCAPH |  |  | 2104 | -0.652 | -0.7376 | Yes |
| 72 | PSMC3IP |  |  | 2106 | -0.659 | -0.7335 | Yes |
| 73 | CKAP2L |  |  | 2109 | -0.663 | -0.7298 | Yes |
| 74 | AURKA |  |  | 2110 | -0.673 | -0.7250 | Yes |
| 75 | ALYREF |  |  | 2111 | -0.677 | -0.7203 | Yes |
| 76 | RPA3 |  |  | 2112 | -0.678 | -0.7155 | Yes |
| 77 | CKS2 |  |  | 2115 | -0.694 | -0.7116 | Yes |
| 78 | CDC20 |  |  | 2117 | -0.695 | -0.7072 | Yes |
| 79 | RAD21 |  |  | 2118 | -0.697 | -0.7023 | Yes |
| 80 | ANP32B |  |  | 2119 | -0.700 | -0.6974 | Yes |
| 81 | CDCA2 |  |  | 2120 | -0.705 | -0.6924 | Yes |
| 82 | HDGF |  |  | 2124 | -0.709 | -0.6889 | Yes |
| 83 | CENPM |  |  | 2125 | -0.709 | -0.6839 | Yes |
| 84 | RAD51AP1 |  |  | 2127 | -0.715 | -0.6794 | Yes |
| 85 | CDCA8 |  |  | 2129 | -0.722 | -0.6748 | Yes |
| 86 | CKAP2 |  |  | 2131 | -0.729 | -0.6701 | Yes |
| 87 | CIP2A |  |  | 2132 | -0.733 | -0.6650 | Yes |
| 88 | ATAD5 |  |  | 2133 | -0.742 | -0.6598 | Yes |
| 89 | TUBA1C |  |  | 2135 | -0.756 | -0.6549 | Yes |
| 90 | KIF2C |  |  | 2136 | -0.756 | -0.6496 | Yes |
| 91 | BRCA1 |  |  | 2138 | -0.758 | -0.6448 | Yes |
| 92 | KIF14 |  |  | 2141 | -0.773 | -0.6403 | Yes |
| 93 | NDC80 |  |  | 2142 | -0.775 | -0.6348 | Yes |
| 94 | KIF23 |  |  | 2145 | -0.782 | -0.6303 | Yes |
| 95 | GMNN |  |  | 2146 | -0.785 | -0.6248 | Yes |
| 96 | LMNB2 |  |  | 2147 | -0.786 | -0.6193 | Yes |
| 97 | MELK |  |  | 2148 | -0.786 | -0.6137 | Yes |
| 98 | ANLN |  |  | 2149 | -0.797 | -0.6081 | Yes |
| 99 | KIFC1 |  |  | 2151 | -0.802 | -0.6030 | Yes |
| 100 | DEPDC1 |  |  | 2152 | -0.809 | -0.5973 | Yes |
| 101 | LSM5 |  |  | 2159 | -0.835 | -0.5943 | Yes |
| 102 | TUBB4B |  |  | 2162 | -0.843 | -0.5894 | Yes |
| 103 | ECT2 |  |  | 2163 | -0.846 | -0.5834 | Yes |
| 104 | HMGB3 |  |  | 2164 | -0.852 | -0.5774 | Yes |
| 105 | KIF20B |  |  | 2165 | -0.852 | -0.5714 | Yes |
| 106 | SPC24 |  |  | 2166 | -0.853 | -0.5654 | Yes |
| 107 | ARHGAP11A |  |  | 2167 | -0.855 | -0.5594 | Yes |
| 108 | DTYMK |  |  | 2168 | -0.858 | -0.5534 | Yes |
| 109 | BUB1 |  |  | 2170 | -0.860 | -0.5478 | Yes |
| 110 | FOXM1 |  |  | 2171 | -0.861 | -0.5418 | Yes |
| 111 | CDCA5 |  |  | 2172 | -0.866 | -0.5357 | Yes |
| 112 | LBR |  |  | 2173 | -0.870 | -0.5296 | Yes |
| 113 | BUB1B |  |  | 2174 | -0.871 | -0.5235 | Yes |
| 114 | PIMREG |  |  | 2175 | -0.876 | -0.5173 | Yes |
| 115 | NUF2 |  |  | 2176 | -0.877 | -0.5111 | Yes |
| 116 | CEP55 |  |  | 2177 | -0.883 | -0.5049 | Yes |
| 117 | KPNA2 |  |  | 2178 | -0.894 | -0.4987 | Yes |
| 118 | HMMR |  |  | 2179 | -0.895 | -0.4924 | Yes |
| 119 | CCDC34 |  |  | 2180 | -0.898 | -0.4861 | Yes |
| 120 | CCNA2 |  |  | 2181 | -0.902 | -0.4797 | Yes |
| 121 | MIS18BP1 |  |  | 2183 | -0.903 | -0.4738 | Yes |
| 122 | KIF15 |  |  | 2185 | -0.915 | -0.4679 | Yes |
| 123 | KIF4A |  |  | 2186 | -0.921 | -0.4614 | Yes |
| 124 | PRR11 |  |  | 2187 | -0.930 | -0.4549 | Yes |
| 125 | ORC6 |  |  | 2188 | -0.937 | -0.4483 | Yes |
| 126 | C21orf58 |  |  | 2189 | -0.937 | -0.4417 | Yes |
| 127 | SGO1 |  |  | 2190 | -0.942 | -0.4351 | Yes |
| 128 | CENPH |  |  | 2191 | -0.943 | -0.4285 | Yes |
| 129 | CENPW |  |  | 2192 | -0.943 | -0.4219 | Yes |
| 130 | LMNB1 |  |  | 2194 | -0.950 | -0.4157 | Yes |
| 131 | HJURP |  |  | 2195 | -0.962 | -0.4089 | Yes |
| 132 | UBE2T |  |  | 2196 | -0.965 | -0.4021 | Yes |
| 133 | FANCI |  |  | 2198 | -0.966 | -0.3958 | Yes |
| 134 | CDKN3 |  |  | 2199 | -0.979 | -0.3889 | Yes |
| 135 | CCNB2 |  |  | 2200 | -0.983 | -0.3820 | Yes |
| 136 | CKS1B |  |  | 2201 | -0.987 | -0.3751 | Yes |
| 137 | SGO2 |  |  | 2203 | -1.003 | -0.3685 | Yes |
| 138 | ESCO2 |  |  | 2204 | -1.004 | -0.3614 | Yes |
| 139 | SMC2 |  |  | 2205 | -1.006 | -0.3544 | Yes |
| 140 | KIF11 |  |  | 2206 | -1.008 | -0.3473 | Yes |
| 141 | EZH2 |  |  | 2207 | -1.017 | -0.3401 | Yes |
| 142 | SPC25 |  |  | 2208 | -1.019 | -0.3330 | Yes |
| 143 | CENPE |  |  | 2211 | -1.036 | -0.3266 | Yes |
| 144 | DHFR |  |  | 2212 | -1.040 | -0.3193 | Yes |
| 145 | KNL1 |  |  | 2213 | -1.054 | -0.3119 | Yes |
| 146 | NUDT1 |  |  | 2214 | -1.054 | -0.3045 | Yes |
| 147 | MYBL2 |  |  | 2215 | -1.058 | -0.2971 | Yes |
| 148 | CLSPN |  |  | 2216 | -1.060 | -0.2896 | Yes |
| 149 | CENPN |  |  | 2217 | -1.079 | -0.2820 | Yes |
| 150 | UBE2C |  |  | 2218 | -1.081 | -0.2744 | Yes |
| 151 | CENPU |  |  | 2219 | -1.088 | -0.2668 | Yes |
| 152 | NCAPG |  |  | 2222 | -1.122 | -0.2599 | Yes |
| 153 | H2AFX |  |  | 2223 | -1.146 | -0.2518 | Yes |
| 154 | CCNB1 |  |  | 2224 | -1.148 | -0.2437 | Yes |
| 155 | CDK1 |  |  | 2225 | -1.153 | -0.2356 | Yes |
| 156 | PTTG1 |  |  | 2226 | -1.198 | -0.2272 | Yes |
| 157 | ANP32E |  |  | 2227 | -1.200 | -0.2188 | Yes |
| 158 | MAD2L1 |  |  | 2228 | -1.204 | -0.2103 | Yes |
| 159 | UBE2S |  |  | 2229 | -1.205 | -0.2018 | Yes |
| 160 | GTSE1 |  |  | 2230 | -1.220 | -0.1933 | Yes |
| 161 | DBF4 |  |  | 2232 | -1.247 | -0.1850 | Yes |
| 162 | RRM2 |  |  | 2234 | -1.268 | -0.1766 | Yes |
| 163 | DLGAP5 |  |  | 2235 | -1.273 | -0.1676 | Yes |
| 164 | SMC4 |  |  | 2236 | -1.281 | -0.1586 | Yes |
| 165 | PBK |  |  | 2237 | -1.325 | -0.1493 | Yes |
| 166 | ATAD2 |  |  | 2238 | -1.381 | -0.1396 | Yes |
| 167 | TMPO |  |  | 2239 | -1.382 | -0.1299 | Yes |
| 168 | PRC1 |  |  | 2240 | -1.397 | -0.1200 | Yes |
| 169 | HMGB2 |  |  | 2241 | -1.469 | -0.1097 | Yes |
| 170 | BIRC5 |  |  | 2242 | -1.469 | -0.0994 | Yes |
| 171 | ASPM |  |  | 2244 | -1.490 | -0.0894 | Yes |
| 172 | PCLAF |  |  | 2245 | -1.516 | -0.0787 | Yes |
| 173 | TOP2A |  |  | 2246 | -1.562 | -0.0678 | Yes |
| 174 | NUSAP1 |  |  | 2247 | -1.600 | -0.0565 | Yes |
| 175 | TPX2 |  |  | 2248 | -1.609 | -0.0452 | Yes |
| 176 | CENPF |  |  | 2249 | -1.691 | -0.0333 | Yes |
| 177 | MKI67 |  |  | 2252 | -1.892 | -0.0210 | Yes |
| 178 | HIST1H4C |  |  | 2253 | -2.984 | -0.0000 | Yes |
Table: GSEA details [plain text format]

  

Fig 2: DOROSZKO\_U3013MG\_CLUSTER\_2: Random ES distribution      
 Gene set null distribution of ES for **DOROSZKO\_U3013MG\_CLUSTER\_2**
